# Visible light photoactivity of Polypropylene coated Nano-TiO_2_ for dyes degradation in water

**DOI:** 10.1038/srep17801

**Published:** 2015-12-02

**Authors:** R. Giovannetti, C. A. D’ Amato, M. Zannotti, E. Rommozzi, R. Gunnella, M. Minicucci, A. Di Cicco

**Affiliations:** 1 School of Science and Technology, Chemistry Division, University of Camerino, 62032 Camerino (MC), Italy; 2 School of Science and Technology, Physics Division, University of Camerino, 62032 Camerino (MC), Italy

## Abstract

The use of Polypropylene as support material for nano-TiO_2_ photocatalyst in the photodegradation of Alizarin Red S in water solutions under the action of visible light was investigated. The optimization of TiO_2_ pastes preparation using two commercial TiO_2_, Aeroxide P-25 and Anatase, was performed and a green low-cost dip-coating procedure was developed. Scanning electron microscopy, Atomic Force Microscopy and X-Ray Diffraction analysis were used in order to obtain morphological and structural information of as-prepared TiO_2_ on support material. Equilibrium and kinetics aspects in the adsorption and successive photodegradation of Alizarin Red S, as reference dye, are described using polypropylene-TiO_2_ films in the Visible/TiO_2_/water reactor showing efficient dyes degradation.

Ground water contamination causes a negative effect on water quality and is likely to be the primary source of human contact with toxic chemicals derived from different sources. Organic dyes, undesirable in water, even in very small amounts, are the first pollutant species to be identified due to their obvious color that is aesthetically unpleasant and it can affect the ecosystems integrity^1^. Wastewaters generated by the textile industries contain significant amounts of non-fixed dyes and a huge amount of inorganic salts^2^. For this reasons, degradation and lowering of toxicity of dyes effluents are more important. Because of their complex structure, it’s more difficult to destroy these contaminants which are intentionally chosen to withstand to light and to aerobic oxidation by microorganisms; in fact, only the incomplete degradation of dyes is possible using these processes (physical and microbiological) with the formation of volatile carcinogenic compound as by-product^3^. The preservation of clean air, soil, and water requires the treatment of these dye effluents^3^. In recent years a great deal of interest to the photocatalytic studies regarding organic water pollutants on semiconductor materials emerged^4^,^5^.

The most popular photocatalyst is TiO_2_ semiconductor for its excellent optical and electronic properties, low price, chemical, thermal and biological resistance, no toxicity, recovery and transparency to visible light^6^,^7^,^8^,^9^. For these reasons, TiO_2_ is widely used in many fields such as medical treatment and microorganism disinfection^10^,^11^, dye sensitized solar cells^6^,^8^,^12^, self-cleaning materials^7^,^8^, purification of water, air and also in the solar water splitting^8^. The physical features of TiO_2_ as porosity, morphology, crystallization, surface area and phase transformation influence the photocatalytic activity and the adsorption of the pollutants^13^. In this contest, also different adsorptive materials including silica, perlite, activated carbon, glass^9^,^14^ and others has been tested. The light source plays a primary role in the photocatalytic process due to the fact that the UV or visible irradiation permit different mechanisms. Since TiO_2_ has a large band gap of 3.0–3.3 eV^12^, only the photocatalytic process under the action of UV light is possible because TiO_2_ absorbs a small part of solar spectrum. When TiO_2_ is irradiated by UV-light with higher energy than its band-gap, the semiconductor is excited and an electron injection occurs from the valence band (VB) to the conduction band (CB) in order to create an electron-hole pair (e^−^_CB_, h^+^_VB_). The photogenerated holes diffuse to the semiconductor surface and react with adsorbed water molecules in order to generate hydroxyl radicals (•OH) which can oxidize organic molecules on TiO_2_ surface. Furthermore, the electrons in the conduction band are involved in the reduction process with the air oxygen to produce superoxide radical anions (O_2_^•−^) continuing the photodegradation process^6^,^15^.

Under visible light, only the adsorbed dye on semiconductor surface is excited and an electron injection into the CB of TiO_2_ from the excited dye occurs; this one is converted in cationic dye radical whereas the injected electron (e^−^_CB_) reacts with the pre-adsorbed oxygen producing several forms of radicals.

The overall process are describe as follows^15^,^16^:


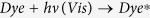



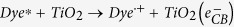



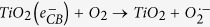



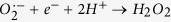



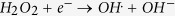



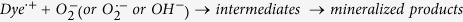


In the photocatalytic process, TiO_2_ can be used in two different ways: immobilized on inert support materials or suspended in aqueous medium. Considering the practical application, immobilized TiO_2_ is preferable than TiO_2_ dispersion to avoid the subsequent steps for the recovery of catalyst after the process. The suspended systems give higher degradation rates but the catalysts must be filtered and, as a result, the process is more expensive^17^. Inert support materials are classified according to their chemical nature; the effects on the semiconductor are the increase of the surface area, change of hydrophobicity, hydrolysis, thermal and chemical stability^5^.

In this study, two commercial types of TiO_2_ such as Anatase (A) and Aeroxide P-25 (P-25) were chosen for the preparation of pastes used to cover Polypropylene (PP) strips. New heterogeneous materials were realized for the photodegradation of Alizarin Red S (ARS) as target pollutants under the action of visible light, in different experimental conditions. Crystallographic and morphological aspects of the new material were studied and SEM, AFM and XRD results are presented.

The work proceeds with the adsorption study of dye on TiO_2_ surface in real time mode and the subsequently photocatalytic degradation process was made in order to obtain equilibrium and kinetic results.

## Results

### PP@TiO_2_ Morphology

**Figure 1:**
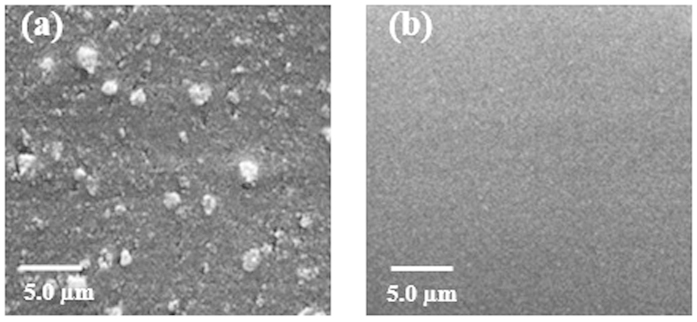
SEM micrographs of as-prepared [PP@TiO_2_] strips. (**a**) [PP@TiO_2_]_P25_ strips before and (**b**) after acidic washing.

Morphological studies by SEM of [PP@TiO_2_]_P25_ before and after acid washing, reported in Fig. 1 show that, the non –regular surface, with the presence of granules excess change to a more regular and uniform layer after this process. From these considerations the acidic treatment is necessary to remove TiO_2_ surplus on PP surface.

**Figure 2:**
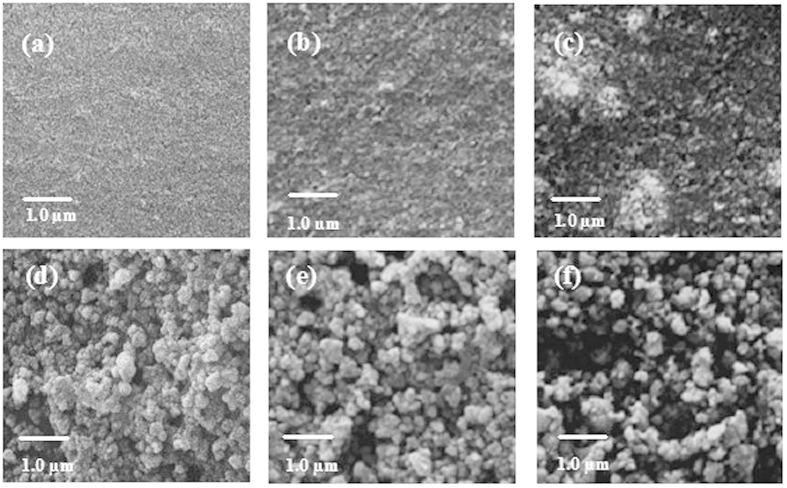
SEM images of as-prepared [PP@TiO_2_] strips and their changes during the studied processes. (**a–c**) SEM images show the change of morphology on as-prepared [PP@TiO_2_]_P25_, after the ARS adsorption and photodegradation. Respectively (**d–f**) SEM images show the change of morphology of as prepared [PP@TiO_2_]_A_, after the dye adsorption and photodegradation respectively.

[PP@TiO_2_]_P25_ and [PP@TiO_2_]_A_ surfaces prepared in the same conditions (Fig. 2a,d), at the same magnification, show morphological differences: [PP@TiO_2_]_P25_ surface presents very small TiO_2_ particles with a homogeneous structures with respect to [PP@TiO_2_]_A_ surface that show instead larger particles of agglomerated nanoparticles.

After ARS adsorption and photodegradation process, changes of PP@TiO_2_ morphology are observed (Fig. 2b,c,e,f). After the adsorption of ARS, both the samples show not homogenous distribution of the material compared to the same sample in the absence of dye while, after the photo-degradation process was observed a decrease of homogeneity of the TiO_2_ surface, due to the loss of the absorbed dye.

**Figure 3:**
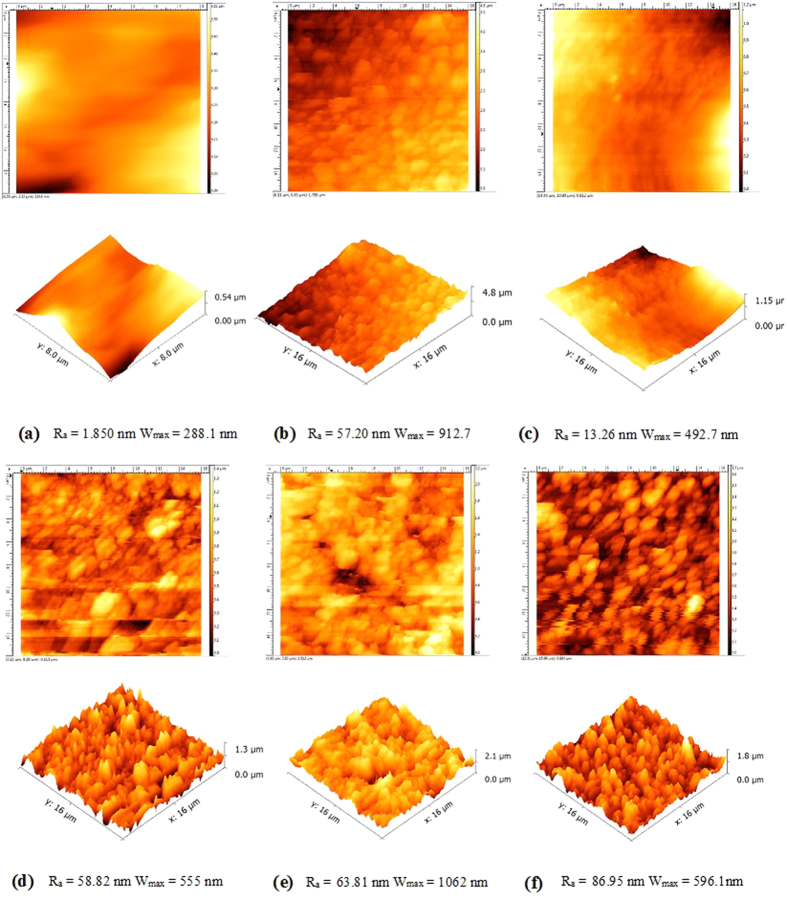
AFM three dimensional surface images. (**a–c**) [PP@TiO_2_]_P25_ films before adsorption, after adsorption and after photodegradation step of ARS respectively; (**d–f**) [PP@TiO_2_]_A_ films before adsorption after adsorption and after photodegradation step of ARS respectively.

AFM was used in order to investigate the topography of TiO_2_ surface, to check the morphological aspects of TiO_2_ layer by surface roughness analysis by several parameters such as root mean square (R_q_), mean roughness (R_a_) and width of ondulation (W_max_) as result of the different processes. Figure 3 shows three dimensional surface images for PP@TiO_2_ films before and after the ARS-adsorption and after the photodegradation step. As well as SEM measurements, AFM images show important differences between [PP@TiO_2_]_P25_ and [PP@TiO_2_]_A_. All R_a_ and W_max_ values of PP@TiO_2_ films without dye, with ARS coated and after the photodegradation step are reported in Fig. 3. [PP@TiO_2_]_P25_, before the dye adsorption, presents smooth surface and it is supposed that this is due to the presence of regular distribution of the particles as can be seen from the SEM images. On the contrary, [PP@TiO_2_]_A_ film present a granular structure and a greater undulating surface respect to [PP@TiO_2_]_P25_. However, as the roughness is caused by the granular structure, which also provides high surface area, it is supposed that [PP@TiO_2_]_A_ layers could exhibit higher adsorption capacity compared to [PP@TiO_2_]_P25_. After adsorption process, the roughness value of [PP@TiO_2_]_P25_ increases considerably due to the dye molecule which cover the smooth surface of [PP@TiO_2_]_P25_.

At the same adsorption conditions, from the R_a_ values of [PP@TiO_2_]_A_, it is possible to suppose that dyes molecules fill the space between holes present on TiO_2_ surface and, as a result, roughness is not modified. The undulating values increase in the adsorption step on [PP@TiO_2_] surface for both Anatase and Aeroxide P-25; after photodegradation step, the average width of undulating values of [PP@TiO_2_]_P25_ and [PP@TiO_2_]_A_ tends to returns to initial conditions, while an increase of average roughness it has been observed only for [PP@TiO_2_]_A_.

In order to reveal structural variations of the compounds, we also collected some powder x-ray diffraction (XRD) patterns before and after the adsorption process.

**Figure 4:**
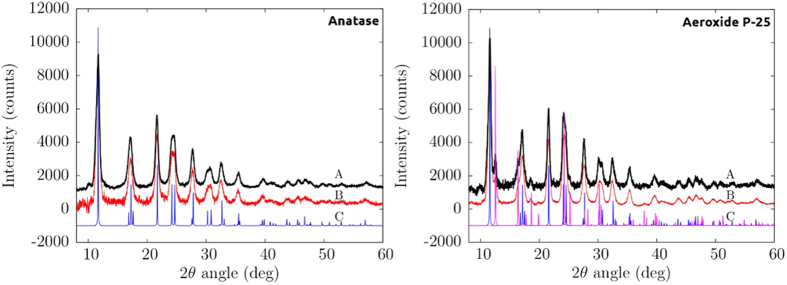
XRD patterns of TiO_2_ Anatase (left side) and Aeroxide P-25 (right side) before (black) and after (red) films preparation.

Figure 4 shows the XRD patterns of TiO_2_ Anatase (left side) and Aeroxide P-25 (right side). In this figure the black line (A) and the red line (B) are referred respectively to the data collected before and after the adsorption process, while are also visible the calculated patterns for Anatase (blue) and Rutile (magenta) structure phases (C).

**Figure 5:**
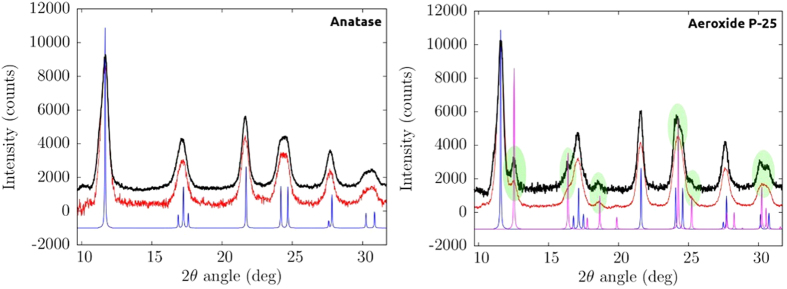
Magnification in 10–32 deg 2θ range of TiO_2_ Anatase (left side) and Aeroxide P-25 (right side) before (black) and after (red) films preparation. The green shaded areas give evidence to the peaks associated with the Rutile phase.

In Fig. 5 we present a magnification of our XRD data in the range 10–32 deg in order to better analyze the main diffraction peaks.

The analysis of the [PP@TiO_2_]_A_ pattern reveals that the structure of TiO_2_ (Anatase) remains unchanged after pastes preparation, indicating that the process did not modify the characteristic nanocrystal structure of TiO_2_. The comparison of [PP@TiO_2_]_P25_ (mixture of Anatase and Rutile phases) patterns shows any changement of the Anatase diffraction lines, while the peaks assigned to the Rutile phase are slightly more pronounced, well visible in the Fig. 5 (right panel) in the green shaded areas. Growth and sharpening peaks indicate a weak structural reorganization effects of the structure of the nanocrystalline Rutile phase.

### Equilibrium and Kinetic Studies of ARS Adsorption

**Figure 6:**
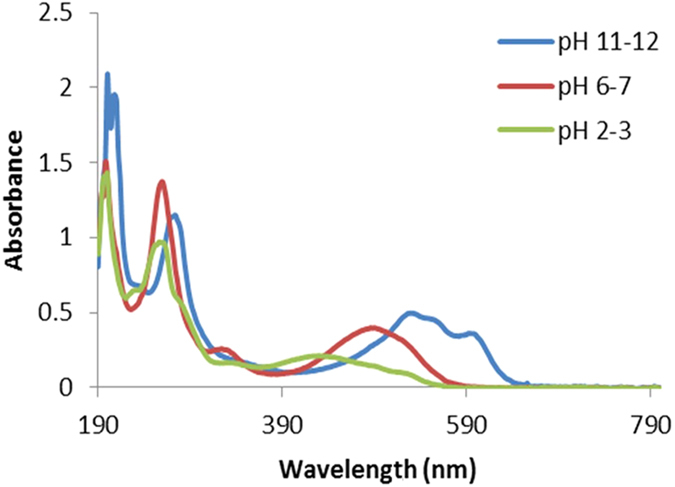
UV-Vis spectra of ARS at different pH.

ARS solution, at acidic pH (2–3), is yellow and present characteristic UV-Vis spectrum with two predominant bands at 261 and 424 nm. Increasing the pH up to 6–7, the bands at 424 nm shift to 510 nm with an increase of the molar extinction coefficient and the solution is strong orange. Fine structure with the presence of two bands at 556 and 596 nm is obtained at basic pH (11–12); in this case the solution is violet and the bands located around 260 nm can be assigned to the π–π* transition of anthraquinone structure (Fig. 6)^18^.

**Figure 7:**
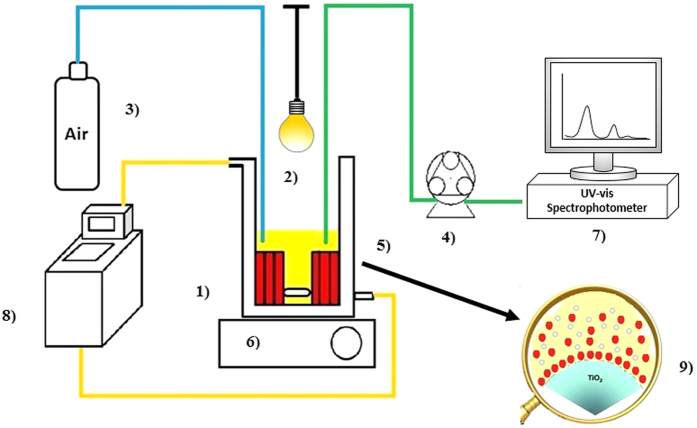
Scheme of photoreactor. (1) cylindrical thermostated glass vessel; (2) visible lamp; (3) air flux; (4) peristaltic pump Gilson miniplus 3; (5) support coated TiO_2_; (6) magnetic stirred; (7) UV-Vis spectrophotometer; (8) thermostat; 9) magnification of TiO_2_-dye interaction.

The ARS adsorption on TiO_2_ surface in dark condition at 25 °C was first studied using the photoreactor showed in Fig. 7. According to the literature data^19^, the pH is the major factor that influence the photocatalytic process because affects the adsorption of dye molecules onto TiO_2_ surface. The adsorption study of ARS on PP@TiO_2_ conducted at different pH showed that the acidic condition gives the better adsorption efficiency. In fact, the pH condition influences the charge properties of TiO_2_ and, for higher pH than the point of zero charge (pzc) that, for Aeroxide P-25 is at pH 6.5, the surface becomes negatively charged and it is the opposite for pH < pzc, according to the reaction^1^,^20^.


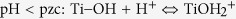



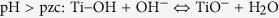


The same acidic condition influences also the chemistry of dye; in fact, ARS structure shows a negative charge on sulphonic group^20^ and, with pH < 6, an evident adsorption of dye was observed as a result of the electrostatic attraction of the positively charged TiO_2_ with ARS dye.

**Figure 8:**
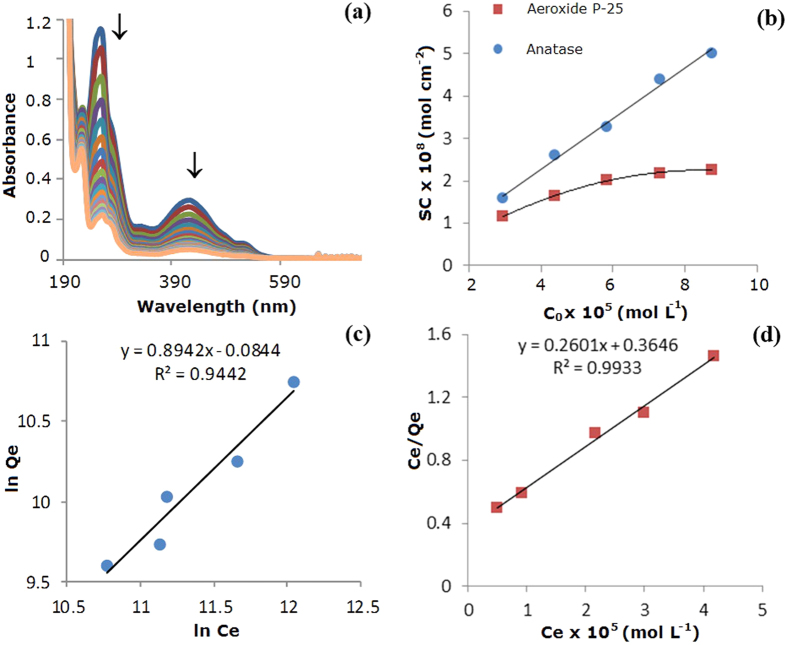
Decrease of UV-Vis spectra in the solution during the adsorption process of 7.30 × 10^−5^ mol L^−1^ of ARS on [PP@TiO_2_]_A_ (**a**); surface ARS concentration (SC) increasing ARS solution concentrations from 2.92 × 10^−5^ to 8.77 × 10^−5^ mol L^−1^ on [PP@TiO_2_]_P25_ (red squares) and [PP@TiO_2_]_A_ (blue dots) (**b**); isotherm graphs for ARS on [PP@TiO_2_]_A_ (**c**) and on [PP@TiO_2_]_P25_ (**d**).

Colour change of adsorbed dyes (red) compared to that of the initial dye solutions (yellow) suggest in fact the neutralization of dye charges on the TiO_2_ surface. It is therefore possible to suppose that with high adsorption capacity of photoactive titanium dioxide, it is possible to improve the efficiency of photo-degradation. For this, to obtain a good knowledge of ARS adsorption mechanism and in order to evaluate how the dye concentration in the original solution influences the adsorption capacity of PP@TiO_2_ films, different starting ARS concentrations in the adsorption process have been used. Figure 8a shows the UV-Vis spectra change in the solution during the adsorption of ARS on [PP@TiO_2_]_A_.

A common method for defining adsorption equilibrium is the analysis of the “adsorption isotherms”. The surface concentration on TiO_2_ surface can be calculated by the equation 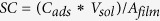 where *C*_*ads*_ is the adsorbed ARS concentration (mol L^−1^), *V*_*sol*_ is the volume of ARS solution (L) and *A*_*film*_ is equal to 270 cm^2^ considering the total area of 9 PP strips.

Figure 8b shows the growth of surface concentration *SC* (mol cm^−2^) of ARS on PP@TiO_2_ films increasing ARS solution concentrations. The [PP@TiO_2_]_A_ surface had greater absorption capacity compared to that obtained with [PP@TiO_2_]_P25_ that showed instead an absorption near to the saturation point of all active sites on TiO_2_ surface. To establish as the dye concentration influences the adsorption process, the equilibrium data has been analyzed by linear forms of Freundlich (1) and Langmuir (2) isotherms^21^:


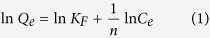



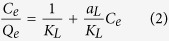


where *C*_*e*_ is the dye solution concentration (mol L^−1)^, *Q*_*e*_ is the adsorbed dye amount on TiO_2_ at equilibrium (mol L^−1^), *K*_*F*_ is the Freundlich constant that represents the adsorption capacity, 1/*n* is the adsorption intensity, *K*_*L*_ and *a*_*L*_ are the Langmuir constant and *K*_L_/*a*_*L*_ represent the theoretical saturation capacity of the TiO_2_ surface, *Q*_*0*_.

In Fig. 8c,d were reported the linearized isotherm plots that were used to calculate the adsorption constants. The results show that the adsorption data of [PP@TiO_2_]_A_ fitting Freundlich isotherm model with *K*_*F*_ of 9.19 × 10^−1^ and *1/n* of 0.89 (correlation coefficient of 0.944) while for the [PP@TiO_2_]_P25_ the best fit is given by the Langmuir model with *K*_*L*_ of 2.74, *Q*_*0*_ of 3.84 × 10^−5^ and *a*_*L*_ of 7.13 × 10^4^ (correlation coefficient of 0.989).

According to the Langmuir model it may be deduced that, in the adsorption process on [PP@TiO_2_]_P25_, all dye molecules incorporated into the film have similar adsorption energy, and the maximum ARS adsorption corresponds to a saturated layer of dye molecules on the adsorbing TiO_2_ surface that cannot contribute to an additional incorporation of other molecules. However, according to the Freundlich model, it may be deduced that, in the adsorption process on [PP@TiO_2_]_A_, multilayer adsorption mechanism can occur^22^.

These behaviours are explained because [PP@TiO_2_]_P25_ presents a regular and uniform structure of the surface, as demonstrated by SEM and AFM measurements and therefore the dye chemisorption is favoured; [PP@TiO_2_]_A_ shows instead a highly rough surface which permits also high multilayer physical-sorption of dye. These different features explain the greater absorption capacity of [PP@TiO_2_]_A_ surface respect to [PP@TiO_2_]_P25_.

To further characterize the kinetic of adsorption process, the experimental data were compared with different kinetic models and it has been found that a pseudo-first order kinetic well describe the obtained results. The pseudo first order kinetic is expressed by the following equation:


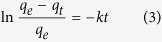


where *q*_*t*_ is the amount of adsorbed dye at time *t*, *q*_e_ is the equilibrium concentration and *k* is the first-order adsorption rate constant.

**Figure 9:**
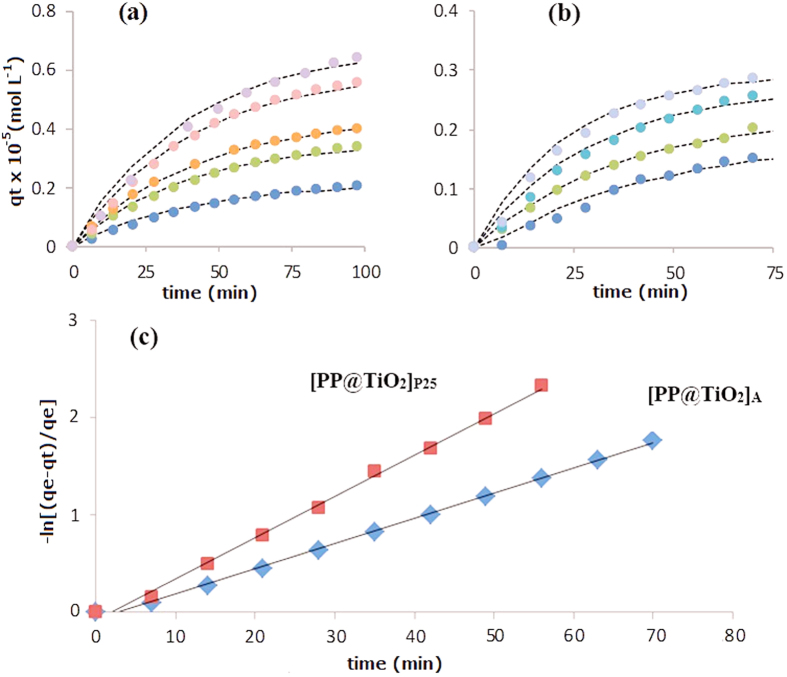
Time evolution of *q*_*t*_ for different initial ARS concentrations for both [PP@TiO_2_]_A_ (**a**) and [PP@TiO_2_]_P25_ (**b**) respectively; pseudo first order adsorption kinetic of ARS (7.30 × 10^−5^ mol L^−1^) in acidic conditions using [PP@TiO_2_]_A_ and [PP@TiO_2_]_P25_ respectively (**c**).

Figure 9a,b show the variation over time of adsorbed dye for different starting ARS concentrations for both [PP@TiO_2_]_A_ and [PP@TiO_2_]_P25_ respectively. The plot of the logarithm term equation (3) versus time t gives optimal straight lines where the slope corresponds to the *k* value as reported in Fig. 9c for the adsorption of ARS on [PP@TiO_2_]_A_ and [PP@TiO_2_]_P25_ respectively.

The obtained adsorption kinetic constant for [PP@TiO_2_]_A_ shows that *k*_A_ does not change for different initial dye concentrations showing mean value of (2.54 ± 0.03) × 10^−2^ min^−1^.

However, for [PP@TiO_2_]_P25_ film, *k*_D_ shows a linear dependence on initial dye concentration as may be seen in the Table 1.

## Photodegradation Process

ARS solutions in absence of PP@TiO_2_ are stable under visible light irradiation but, after the adsorption process, the signal of ARS band decreased up to complete disappearance while the colour of PP@TiO_2_ changes from red to colourless, indicating the photodegradation of ARS with the destruction of their characteristic chromophore; no new adsorption bands appeared in the UV-Vis region.

**Figure 10:**
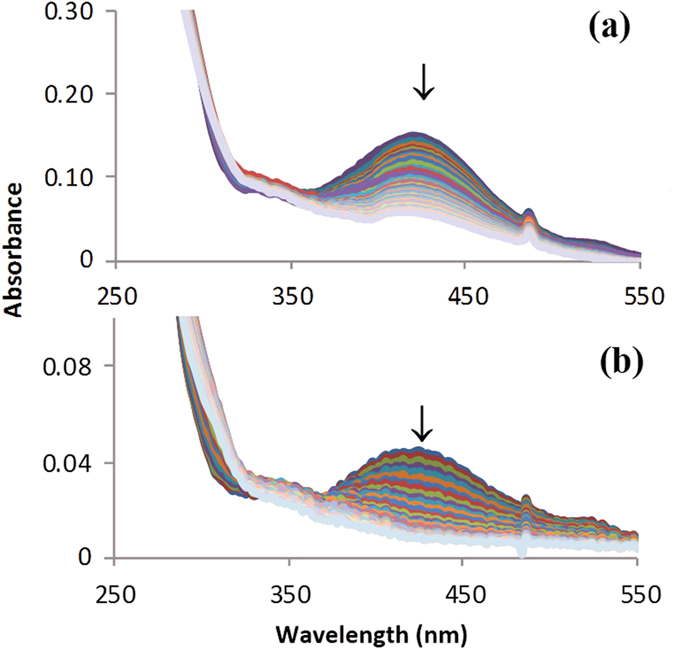
Spectral change in the solution, every 7 minutes, during the photodegradation step under visible irradiation of ARS (7.30 × 10^−5^ mol L^−1^) in acidic conditions, for (**a**) [PP@TiO_2_]_P25_ and (**b**) [PP@TiO_2_]_A_ respectively.

The photodegradation step, under visible light irradiation, monitored during time by the UV-Vis spectra profile in solution at 424 nm (Fig. 10), demonstrate that this process is slower with respect to the adsorption process, indicating that the reaction occurs on TiO_2_ surface and not in the solution where the absorbance values are real time monitored.

The variation in time of dye concentration Uv-Vis spectra illustrates that the photodegradation process follows pseudo first order kinetics with respect to dye concentration as shown in equation (4):


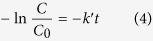


where *k′* is the pseudo first order kinetic constant, *C*_*0*_ is the initial concentration of dye and *C* the concentration of dye at t time.

**Figure 11:**
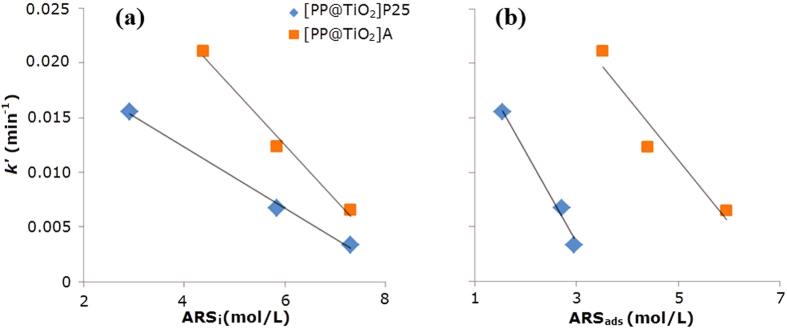
Relation between *k*’ with (**a**) initial and (**b**) adsorbed for both [PP@TiO_2_]_P25_ and [PP@TiO_2_]_A_ respectively.

The obtained rate constants values *k′*_*A*_ and *k′*_*P25*_ (Table 2) linearly decrease with the increase of initial dye concentration (ARS_i_), and with adsorbed dye concentration (ARS_ads_) as reported in Fig. 11, showing the influence of catalyst loading in the overall process.

**Figure 12:**
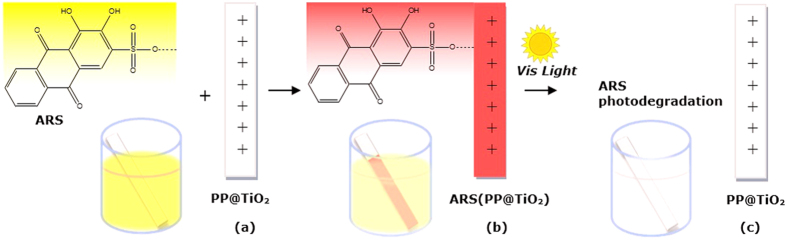
Schematic adsorption and Visible-photodegradation of ARS on TiO_2_. The model shows ARS and TiO_2_ color changes: (**a**) after immersion, (**b**) after adsorption and (**c**) after photodegradation.

**Figure 13:**
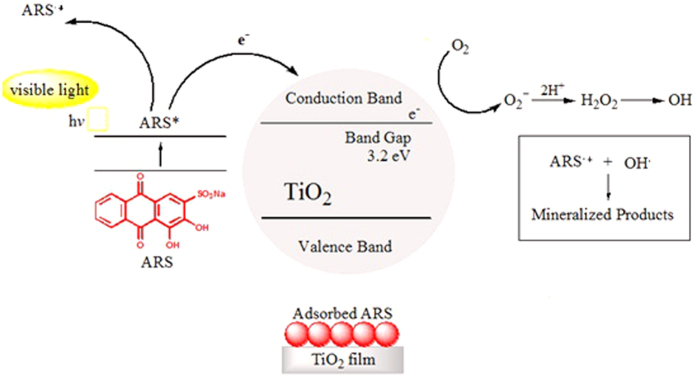
Schematic representation of ARS photodegradation process.

The overall process of adsorption and photodegradation can be resumed in the Fig. 12, while the mechanism is represented schematically in Fig. 13.

## Conclusions

Polypropylene coated nano-TiO_2_ photocatalysts were successfully prepared. The optimization of TiO_2_ pastes preparation using two commercial TiO_2_ has been performed and green, low-cost dip-coating procedure was developed.

The study was conducted in three steps. In the first step, Scanning Electron Microscopy, Atomic Force Microscopy and XRD measurements were used to obtain morphological and structural informations of the new heterogeneous photocatalysts on PP support material. Morphological studies demonstrate that [PP@TiO_2_]_A_ presents major rugosity respect to [PP@TiO_2_]_P25_. The analysis of the [PP@TiO_2_] XRD patterns after pastes preparation reveals that the characteristic nanocrystal structure of TiO_2_ (presents on each photocatalyst) shows no changement of the Anatase diffraction lines while a weak structural reorganization effect of the Rutile phase, present only on [PP@TiO_2_]_P25_, is observed.

In the second step, equilibrium and kinetic aspects of ARS adsorption process were studied showing that the absorption process of ARS followed Freundlich isotherm model with [PP@TiO_2_]_A_ and Langmuir isotherm model with [PP@TiO_2_]_P25_. Obtained *K*_F_ value of [PP@TiO_2_]_A_ is 3.35 times higher respect to [PP@TiO_2_]_P25_ showing that [PP@TiO_2_]_A_ has greater absorption capacity compared to [PP@TiO_2_]_P25_.

The results of adsorption kinetics revealed that ARS adsorption is kinetically favoured from the increase of initial dye concentration using [PP@TiO_2_]_P25_ with kinetic constant value of 2.07 × 10^−2^ that became about double when the concentration increases of 2.5 factor. On the contrary by using [PP@TiO_2_]_A_ the kinetic constant value of 2.54 × 10^−2^ did not change for different initial concentration.

Finally, in the third step, after ARS adsorption in dark condition, kinetic data are obtained from photodegradation process under the action of visible light showing that kinetic constants decrease with the increase of initial ARS concentration for each photocatalyst. However, the results demonstrate that the values obtained by using [PP@TiO_2_]_A_ are about 20 times higher with respect to [PP@TiO_2_]_P25_ at the same initial concentration showing therefore, for this photocatalyst, higher photodegradation activity.

Comparison of the obtained results of [PP@TiO_2_]_A_ with those of [PP@TiO_2_]_P25_, because the differences of two TiO_2_ starting materials derived only from the presence of rutile phase, it is possible to deduce that the rutile phase presents only on [PP@TiO_2_]_P25_ also with a weak structural reorganization, affected the characteristics of adsorption surface and greatly the photodegradation activity.

According to all obtained results it might be confirm that the use of PP@TiO_2_ in the Visible light photodegradation of ARS permits to obtain efficiently dye degradation, with addition advantage of easy separation of photocatalyst and possibility of reuse, suggesting an application for environmental treatment of waste water.

## Methods

### Catalyst pastes preparation

Two type of pastes were prepared using TiO_2_ Aeroxide P-25 and Titanium (IV) dioxide Anatase nano-powdered (<25 nm) (Sigma Aldrich, Code 718467). To create the paste, TiO_2_ powder (6 g) was ground in a porcelain mortar and mixed with 2 ml of distilled water containing 10% v/v of acetyl acetone (Sigma Aldrich) in order to prevent coagulation of TiO_2_ nanoparticles. The paste was diluted further with distilled water (8 ml) with continuous grinding for 3 minutes; after, in order to facilitate the spreading of the pastes on the substrate, few drops of a detergent (Triton X-100 Sigma Aldrich) were added. The final addition of water controls the viscosity and the final concentration of pastes.

### Polypropylene coated Nano-TiO_2_ preparation (PP@TiO_2_)

PP strips (3M Polypropylene 2500 material) of defined size with 2 cm of width and 10 cm of length were prepared and cleaned with ethanol. TiO_2_ paste (Aeroxide P-25 or Anatase) was added in a cylindrical glass until reaching a determinate level and the paste was stratified on PP strips with the dip-coating technique. After each immersion of 1 minute, the strip is extracted, dried at the room temperature and further dried in the oven at the temperature of ~110 °C for 30 minutes. Finally, the strips are clean up with a solution of water and HCl 0.1 N to remove the excess of TiO_2_ particles. This procedure is repeated for 3 times in order to obtain PP coated TiO_2_ strips (PP@TiO_2_) with an adequate thickness.

### Catalyst films characterization

The surface morphology of [PP@TiO_2_]_P25_ (Aeroxyde P-25) and [PP@TiO_2_]_A_ (Anatase), before and after dye adsorption and photocatalytic process, was evaluated by Scanning Electron Microscopy (SEM Leica Cambridge Stereoscan) at an accelerating voltage of 20 kV and by Atomic Force Microscopy (AFM Veeco 5000 Dimension) working in tapping mode. For SEM measurements, the strips were attached on aluminium stabs using self-adhesive carbon conductive tabs and sputter coated with gold by Balzers Med 010.

In order to reveal structural variations of the compounds, we also collected some powder x-ray diffraction patterns before and after the adsorption process.

The XRD measurements were collected on a customized horizontal Debye-Scherrer diffractometer, equipped with a INEL CPS 180 curved position sensitive detector that allow to reduce drastically the acquisition time for each pattern. In fact this detector, filled with an Kr/CO_2_ gas mixture in order to optimize the efficiency, have no moving parts and eliminates the need for mechanical scanning devices such as complex scanning goniometers used in conventional XRD instruments.

The x-ray source is a Mo K-alpha (lambda = 0.7093 Å), generated by a Philips sealed x-ray tube and monochromatized through a graphite crystal along the 002 plane. The samples were positioned on the beam into glass capillaries (diameter 100microns).

### Dye adsorption and photodegradation testing

The adsorption (in dark conditions) and photodegradation (under visible light) processes were investigated at acidic pH (by addition of HCl 1.0 N Sigma-Aldrich) using the photo-reactor (Fig. 6).

The reactor is formed by a cylindrical glass vessel with thermostatic chamber (fixed at 25 °C) containing different starting Alizarin Red S (1,2-dihydroxy-9,10-anthraquinonesulfonic acid sodium salt or ARS) dye solutions from 2.92 to 8.77 × 10^−5^ mol L^−1^, visible lamp (tubular JD, 80 W, 1375 Lumen DURA), air-line, peristaltic pump Gilson miniplus 3, nine strips of PP@TiO_2_ and magnetic stirred. The system has been connected with spectrophotometer Hewlett-Packard 8452°. Diode Array with a continuous flux quartz cuvette of optical path of 1 cm, in order to operate in real-time mode. The adsorption and photodegradation processes were separately monitored by measuring the absorbance spectra of dye solutions every 7 minutes at the wavelength maximum of ARS (424 nm); the decrease in concentration of the dye was plotted as function of time.

## Additional Information

**How to cite this article**: Giovannetti, R. *et al*. Visible light photoactivity of Polypropylene coated Nano-TiO2 for dyes degradation in water. *Sci. Rep*. **5**, 17801; doi: 10.1038/srep17801 (2015).

## Figures and Tables

**Table 1:** Adsorption kinetic constants *k*_(P25)_ for ARS adsorption on [PP@TiO_2_]_P25_ in acidic condition.

[ARS] (mol L^−1^)	*k*_(P25)_ (min^−1^)
2.922 × 10^−5^	(2.07 ± 0.02) × 10^−2^
4.383 × 10^−5^	(2.83 ± 0.03) × 10^−2^
5.844 × 10^−5^	(3.34 ± 0.02) × 10^−2^
7.304 × 10^−5^	(4.25 ± 0.04) × 10^−2^

**Table 2:** Photodegradation kinetic constants for ARS in acidic condition on [PP@TiO_2_]_A_ and [PP@TiO_2_]_P25_ respectively.

[ARS] (mol L^−1^)	*k*′_A_ (min^−1^)	[ARS] (mol L^−1^)	*k*′_P25_(min^−1^)
4.38 × 10^−5^	(2.11 ± 0.03) × 10^−2^	2.92 × 10^−5^	(1.55 ± 0.03) × 10^−2^
5.84 × 10^−5^	(1.23 ± 0.02) × 10^−2^	5.84 × 10^−5^	(0.67 ± 0.02) × 10^−2^
7.30 × 10^−5^	(0.65 ± 0.02) × 10^−2^	7.30 × 10^−5^	(0.33 ± 0.02) × 10^−2^
